# Rare single-nucleotide *DAB1* variants and their contribution to Schizophrenia and autism spectrum disorder susceptibility

**DOI:** 10.1038/s41439-020-00125-7

**Published:** 2020-11-10

**Authors:** Yoshihiro Nawa, Hiroki Kimura, Daisuke Mori, Hidekazu Kato, Miho Toyama, Sho Furuta, Yanjie Yu, Kanako Ishizuka, Itaru Kushima, Branko Aleksic, Yuko Arioka, Mako Morikawa, Takashi Okada, Toshiya Inada, Kozo Kaibuchi, Masashi Ikeda, Nakao Iwata, Michio Suzuki, Yuko Okahisa, Jun Egawa, Toshiyuki Someya, Fumichika Nishimura, Tsukasa Sasaki, Norio Ozaki

**Affiliations:** 1 Department of Psychiatry, Nagoya University Graduate School of Medicine, Nagoya, Aichi Japan; 2 Brain and Mind Research Center, Nagoya University, Nagoya, Aichi Japan; 3 Institute for Advanced Research, Nagoya University, Nagoya, Aichi Japan; 4 Center for Advanced Medicine and Clinical Research, Nagoya University Hospital, Nagoya, Aichi Japan; 5 Department of Cell Pharmacology, Nagoya University Graduate School of Medicine, Nagoya, Aichi Japan; 6 Department of Psychiatry, Fujita Health University School of Medicine, Toyoake, Aichi Japan; 7 Department of Neuropsychiatry, University of Toyama Graduate School of Medicine and Pharmaceutical Sciences, Toyama, Japan; 8 Department of Neuropsychiatry, Okayama University Graduate School of Medicine, Dentistry and Pharmaceutical Sciences, Okayama, Japan; 9 Department of Psychiatry, Niigata University Graduate School of Medical and Dental Sciences, Niigata, Japan; 10 Office for Mental Health Support, Center for Research on Counseling and Support Services, The University of Tokyo, Tokyo, Japan; 11 Department of Physical and Health Education, Graduate School of Education, The University of Tokyo, Tokyo, Japan

**Keywords:** Targeted resequencing, Autism spectrum disorders, Schizophrenia

## Abstract

Disabled 1 (DAB1) is an intracellular adaptor protein in the Reelin signaling pathway and plays an essential role in correct neuronal migration and layer formation in the developing brain. *DAB1* has been repeatedly reported to be associated with neurodevelopmental disorders including schizophrenia (SCZ) and autism spectrum disorders (ASD) in genetic, animal, and postmortem studies. Recently, increasing attention has been given to rare single-nucleotide variants (SNVs) found by deep sequencing of candidate genes. In this study, we performed exon-targeted resequencing of *DAB1* in 370 SCZ and 192 ASD patients using next-generation sequencing technology to identify rare SNVs with a minor allele frequency <1%. We detected two rare missense mutations (G382C, V129I) and then performed a genetic association study in a sample comprising 1763 SCZ, 380 ASD, and 2190 healthy control subjects. Although no statistically significant association with the detected mutations was observed for either SCZ or ASD, G382C was found only in the case group, and *in silico* analyses and in vitro functional assays suggested that G382C alters the function of the DAB1 protein. The rare variants of *DAB1* found in the present study should be studied further to elucidate their potential functional relevance to the pathophysiology of SCZ and ASD.

## Neurodevelopment: Rare mutation associated with schizophrenia and autism

A rare mutation could affect nerve development in the brain, potentially increasing susceptibility to schizophrenia and autism spectrum disorder. Hiroki Kimura of Nagoya University Graduate School of Medicine and colleagues in Japan screened the protein-coding parts of the gene DAB1 in people with schizophrenia or autism for rare nucleotide variations. DAB1 is important for neurodevelopment and synaptic function, and has been associated with neurodevelopmental disorders. Their investigations revealed a mutation that can change the structure and stability of the protein coded by DAB1, potentially increasing susceptibility to neurodevelopmental disorders. The mutation was found in people with schizophrenia and autism, but not in healthy controls. Further research with larger patient samples and investigations on mice engineered to include the mutation could clarify its impact on the pathophysiology of schizophrenia and autism.

## Introduction

Schizophrenia (SCZ) is a devastating psychiatric disorder that is characterized by hallucinations, delusions, and cognitive impairment and causes tremendous individual and societal burden^1^. The lifetime prevalence of SCZ is estimated to be 0.48% in the general population^2^. Autism spectrum disorder (ASD), which affects 1–2% of children, is characterized by deficits in social communications and social interactions and by restricted, repetitive patterns of behavior^3,4^. The heritability of SCZ and ASD is as high as 80%, making these conditions targets for human genetics research^5,6^. Previous studies suggested that these disorders share genetic risk factors^7^.

Recent large-scale genome-wide association analysis^8^, whole-exome sequencing^9,10^, and copy number variation (CNV) analysis^11,12^ performed with SCZ and ASD samples have revealed that deleterious rare genetic variants such as single-nucleotide variants (SNVs) and CNVs exert significantly larger effects than common single-nucleotide polymorphisms (SNPs). Furthermore, rare SNVs discovered from the sequencing of susceptibility genes may have a large effect size and account for a portion of the heritability of SCZ and ASD; they could also contribute to our understanding of the etiopathology of neurodevelopmental disorders following further functional assays^13–16^.

Disabled 1 (*DAB1*) is a gene involved in the Reelin signaling pathway that is related to susceptibility to SCZ and ASD^17–22^. DAB1 is an intracellular adaptor protein in the Reelin signaling pathway and plays an essential role in correct neuronal positioning and layer formation in the developing brain as well as in synaptic function, learning, and memory in the adult brain^23–25^.

The binding of Reelin to its lipoprotein receptors induces DAB1 phosphorylation by the Src family kinases Fyn and Src^26–28^. The signal is then transmitted to downstream molecules such as Crk/CrkL^29–31^, SOCS3^32^, Nckβ^33^, PI3K^34^, and Lis1^35^. These downstream pathways control multiple steps in neuronal migration and modulate synaptic function via regulation of the actin cytoskeleton, cell adhesion molecules, and microtubules^36–38^.

*DAB1-*deficient mice and phospho-mutant *DAB1* mice exhibit *reeler*-like phenotypes^23,39,40^ and show some abnormalities in behavior and brain structure that are similar to the abnormalities observed in certain neurodevelopmental disorders, including SCZ and ASD^41^. Furthermore, dorsal forebrain-specific *DAB1* conditional knockout mice exhibit behavioral abnormalities including hyperactivity, decreased anxiety-like behavior, and impaired working memory, which are symptoms observed in patients with SCZ^42^. A postmortem study showed that *DAB1* mRNA is significantly decreased in the superior frontal and cerebellar areas of autistic brains compared to control brains^43^.

Considering that *DAB1* is a Reelin signaling gene and is strongly associated with neurodevelopment and synaptic function, we hypothesized that rare variants in *DAB1* may contribute to SCZ and ASD susceptibility. However, no whole-exome sequencing studies have shown associations between SCZ or ASD and rare variants in *DAB1*, and no studies have been conducted focusing only on identifying rare *DAB1* variants through deep sequencing of SCZ and ASD samples. Moreover, our earlier CNV analysis of SCZ and ASD did not identify variants in *DAB1* regions^44^. Therefore, to discover rare variants with a large effect size and to explore the role of the discovered rare variants in SCZ and ASD pathogenesis, we performed mutation screening of *DAB1* exons with SCZ and ASD samples, followed by several studies: (1) an association study targeting the discovered rare variants in sample sets of SCZ patients, ASD patients, and healthy controls (CON); (2) *in silico* analyses of the rare variants with putative large effects; and (3) functional analysis to compare the biological stability of the DAB1 wild-type (WT) and mutant proteins. In this study, we found that the rare *DAB1* variant G382C may have functional relevance to the pathophysiology of SCZ and ASD.

## Materials and methods

### Participants

Two independent Japanese sample sets were used in this study (Table 1). The first set, comprising 370 SCZ (mean age = 49.7 ± 14.8 years; males = 53.0%) and 192 ASD (mean age = 16.3 ± 8.4 years; males = 77.6%) patients, was used as the targeted resequencing discovery cohort. The second set, comprising 1763 SCZ (mean age = 47.6 ± 15.3 years; males = 53.1%), 380 ASD (mean age = 21.4 ± 10.5 years; males = 77.6%), and 2190 healthy CON subjects (mean age = 45.7 ± 15.0 years; males = 51.6%), was used for genetic association analysis.

**Table 1:** Profiles of participants in the resequencing and association analysis sample sets.

	Resequencing		Association analysis		
	SCZ	ASD	SCZ	ASD	CON
Total	370	192	1763	380	2190
Males (%)	53.0	77.6	53.1	77.6	51.6
Mean age ± SD^a^ (years)	49.7 ± 14.8	16.3 ± 8.4	47.6 ± 15.3	21.4 ± 10.5	45.7 ± 15.0

*SCZ* schizophrenia, *ASD* autism spectrum disorders, *CON* healthy control, *SD* standard deviation.

^a^Age at recruitment.

All participants were unrelated, lived in mainland Japan, and self-identified as Japanese. All patients fulfilled the criteria listed in the Diagnostic and Statistical Manual of Mental Disorders, Fifth Edition (DSM-5) for SCZ or ASD. CON subjects were selected from the general population and had no history of mental disorders based on questionnaire responses from the subjects provided during the sample inclusion step. Written informed consent was obtained from the participants or from their parents, if the participants were under 20 years old, after the verbal description of this study using the research explanation document. All procedures performed in this study involving human participants were approved by the Ethics Committee of the Nagoya University Graduate School of Medicine. The study was conducted in accordance with the Helsinki Declaration of 1975 and its later amendments or comparable ethical standards.

### Resequencing and data analysis

Genomic DNA was extracted from peripheral blood or saliva samples from each SCZ, ASD, and CON participant using the QIAamp DNA Blood Kit or Tissue Kit (QIAGEN, Hilden, Germany). The quantity of extracted DNA was estimated using the Qubit dsDNA BR Assay Kit (Life Technologies, Carlsbad, CA, USA) on a Qubit 2.0 Fluorometer (Life Technologies) following the manufacturer’s recommended protocol. Custom amplification primers were designed to cover exons and flanking intron regions of the selected gene (Ensembl Transcript ID: ENST00000371236.2; GRCh37.p13) with the Ion AmpliSeq Designer (Thermo Fisher Scientific, Waltham, MA, USA). Sample amplification and equalization were achieved using Ion AmpliSeq Library Kits 2.0 and the Ion Library Equalizer Kit, respectively (Thermo Fisher Scientific). Amplified sequences were ligated to Ion Xpress Barcode Adapters (Thermo Fisher Scientific). Emulsion PCR and subsequent enrichment were performed using the Ion OneTouch Template Kit v2.0 on Ion OneTouch 2 and Ion OneTouch ES instruments, respectively (Thermo Fisher Scientific). The final products were then sequenced on the Ion PGM sequencing platform (Thermo Fisher Scientific). Raw data output from the sequencer with the default settings of call quality ≥20 and read depth ≥10 was uploaded to the Torrent Server (Life Technologies) for variant calling with NCBI GRCh37 as a reference. The resulting VCF files were analyzed by Ingenuity Variant Analysis (QIAGEN) for annotation and visualization. The resulting nucleotide sequence data have been deposited in the DNA Data Bank of Japan (DDBJ) databases (http://www.ddbj.nig.ac.jp) under accession number DRA004490.

To prioritize identified mutations, nonsense mutations, missense mutations, small insertions/deletions, and canonical splice-site variants with an allele frequency of <1% in this sequencing and in selected public databases were chosen from the annotated data. We used the following four public exome databases: Genome Aggregation Database (gnomAD) v2.1.1 (https://gnomad.broadinstitute.org), NHLBI Exome Sequencing Project (ESP) Exome Variant Server (http://evs.gs.washington.edu/EVS/), Japanese Multi Omics Reference Panel (jMorp) (https://jmorp.megabank.tohoku.ac.jp/), and Human Genetic Variation Database (HGVD) (http://www.hgvd.genome.med.kyoto-u.ac.jp). The selected variants were validated with Sanger sequencing. The primer sequences used to validate each variant are shown in Table S1.

After prioritizing the detected mutations, prediction of deleterious effects was performed with *in silico* analytic methods: PolyPhen-2 (http://genetics.bwh.harvard.edu/pph2/)^45^ and SIFT (http://sift.jcvi.org/)^46^. Additional pathogenic variant annotations were obtained from NCBI ClinVar (http://www.ncbi.nlm.nih.gov/clinvar/)^47^ and the Human Gene Mutation Database (HGMD) (http://www.hgmd.cf.ac.uk/ac/index.php)^48^. Localization of functional domains and phosphorylation sites was determined based on the Human Protein Reference Database (HPRD) (http://www.hprd.org/index_html). Evolutionary conservation was evaluated with HomoloGene (http://www.ncbi.nlm.nih.gov/homologene/). The structures of *DAB1* proteins with SNVs were predicted and modeled with I-TASSER (https://zhanglab.ccmb.med.umich.edu/I-TASSER/) and UCSF Chimera (https://www.cgl.ucsf.edu/chimera/). We selected the model with the highest C-score, which represents an estimation of the accuracy of the I-TASSER structure models^49^.

### Genetic association analysis

We performed a Custom TaqMan® SNP Genotyping assay (Applied Biosystems) for each variant. DNA samples were prepared in 384-well microtiter plates with positive and negative controls. PCR and allelic discrimination analyses were conducted using the Genotyping Master Mix and Sequence Detection System, respectively, according to standard protocols (Applied Biosystems). To determine whether each detected sample carried the variant of interest, positive samples were reconfirmed with Sanger sequencing or with at least one additional experiment. Differences in genotype distribution between cases and controls were calculated with Fisher’s exact test (one-tailed) with a threshold of significance set at *p* < 0.05.

We computed the effective sample size and statistical power using the web browser program Genetic Power Calculator, which was created by Purcell et al. (http://pngu.mgh.harvard.edu/~purcell/gpc/)^50^.

### Phenotypic analysis

The clinical features of patients with the variant detected in the present study were examined retrospectively from medical records. Psychiatric comorbidities were diagnosed by experienced psychiatrists according to DSM-5 criteria.

### Plasmid construction

The mammalian expression plasmid for *DAB1* was generated with RT-PCR from a human hippocampus cDNA library (Clontech). *DAB1* was cloned into the pEGFPN3 vector and fused with triple V5-epitope tags. The plasmids in this study were prepared with the QIAGEN Plasmid Midi Kit.

### Cycloheximide (CHX) chase assay

Antibodies against the V5-epitope tag (mouse monoclonal, MCA1360, Bio-Rad), DAB1 (rabbit polyclonal, #3328, Cell Signaling) and GAPDH (rabbit polyclonal, #2118, Cell Signaling; mouse monoclonal, M171-3 3H12, MBL Japan) were purchased commercially. CHX was obtained from Sigma-Aldrich. HEK293FT cells (Invitrogen) were maintained in Dulbecco’s modified Eagle’s medium supplemented with 10% fetal bovine serum at 37 °C in a 5% CO_2_ incubator. The plasmids were transfected into HEK293FT cells with Lipofectamine 3000 (Thermo Fisher) according to the manufacturer’s instructions. Then, the transfected cells were cultured for 48 h, and medium containing 100 μg/ml CHX was added for the last 8, 16, or 24 h of culture, as shown in Fig. 3b, until the cells were harvested. The cells were washed with cold PBS and solubilized in SDS sample buffer. Following SDS-PAGE, separated proteins were transferred onto polyvinylidene difluoride membranes (Millipore). The membranes were blocked with Block Ace (Yukijirushi Corp., Japan), incubated with primary antibodies, and then incubated with Alexa 680- and/or Alexa 800-conjugated secondary antibodies. Specific proteins were visualized and quantitated with Odyssey (LI-COR Biosciences).

## Results

### Resequencing and Data Analysis

**Fig. 1:**
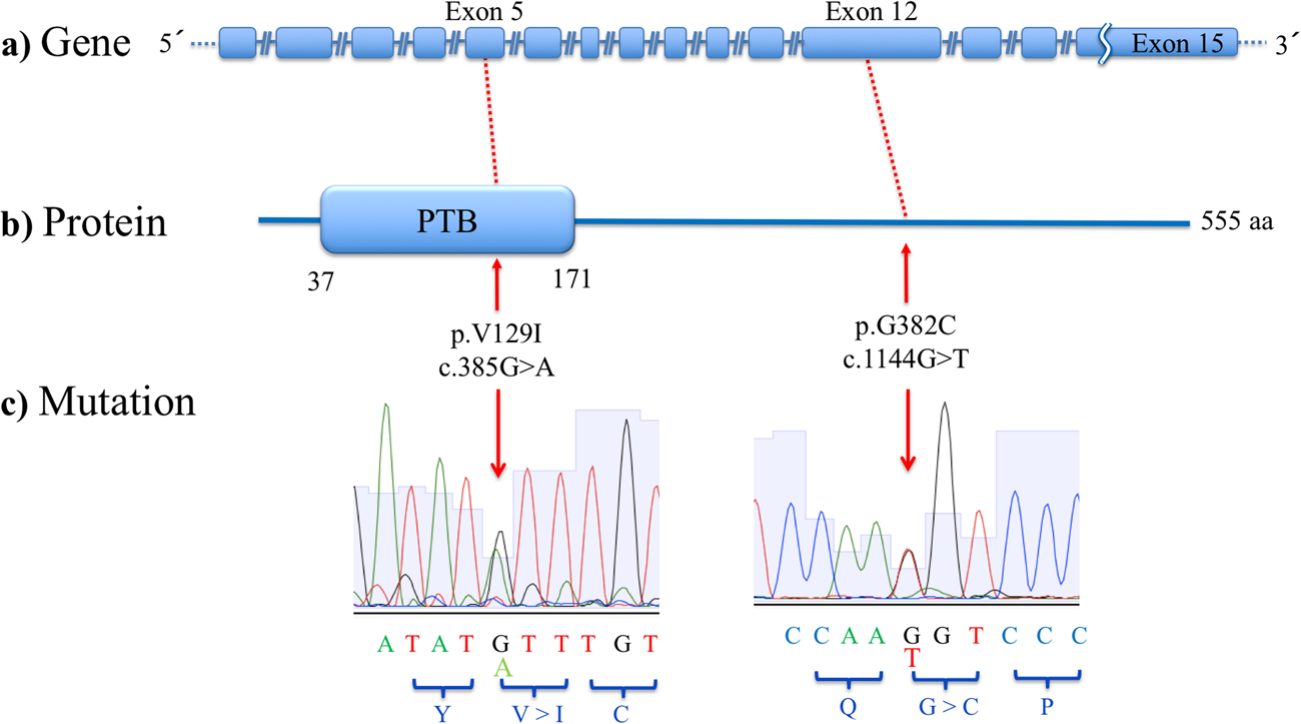
Locations of novel rare variants in *DAB1*. (**a**) The presented *DAB1* structure is based on ENST00000371236.2 (GRCh37.p13). (**b**) The presented protein structure of DAB1 is based on the Human Protein Reference Database. Red dotted lines and arrows indicate the locations of each novel rare variant in DAB1. p.V129I is located in the phosphotyrosine-binding (PTB) domain, and p.G382C is in the C-terminal region. (**c**) Sanger sequencing traces illustrating the novel rare variants. Arrows indicate the mutated sites. The encoded amino acids are shown at the bottom in blue.

To clarify the genetic relationship of *DAB1* with the pathology of SCZ and ASD, we performed mutation screening of *DAB1* coding exons in 370 SCZ and 192 ASD samples. All of the detected variants are described in Table S2. After prioritization of the detected variants, we identified two heterozygous missense variants (p. G382C and p. V129I), which were validated with Sanger sequencing (Fig. 1, Table 2). Nonsense, frameshift, or splice-site mutations were not found. The two missense variants were discovered in one sample each among the 370 SCZ samples. G382C is in the C-terminus, and V129I is located in the phosphotyrosine-binding (PTB) domain, according to HPRD (Fig. 1). G382C was not found in any databases (gnomAD v2.1.1, ESP, jMorp, and HGVD). V129I was rare in gnomAD, jMorp, and HGVD and was not found in ESP (Table 2). None of the SNVs detected in our study were registered in ClinVar or in HGMD.

**Table 2:** Details of discovered rare missense mutations and *in silico* analyses.

Chr	Position^a^	dbSNP ID^b^	Transcript variant	Protein variant	SCZ (*n* = 370)	ASD (*n* = 192)	our cohort MAF^c^	gnomAD^c,d^	ESP^c^	jMorp (4.7KJPN)	HGVD Frequency	*In silico* analysis	
												SIFT	PolyPhen-2
1	57480856	rs915578825	c.1144 G > T	p.G382C	1 F	0	1/1124	–	–	–	–	Damaging	Probably Damaging
1	57538009	rs755758685	c.385 G > A	p.V129I	1 M	0	1/1124	4/251140	–	0.0008	0.0009	Damaging	Probably Damaging

*Chr* Chromosome, *SCZ* schizophrenia, *ASD* autism spectrum disorders, *MAF* minor allele frequency, *gnomAD* Genome Aggregation Database, *ESP* Exome Sequencing Project, *jMorp* Japanese Multi Omics Reference Panel, *HGVD* Human Genetic Variation Database, *F* Female, *M* Male.

^a^Genomic position based on NCBI builds GRCh 37 (Transcript ID ENST00000371236.2).

^b^dbSNP Build 154.

^c^minor allele count/total allele count.

^d^gnomAD v2.1.1.

### Bioinformatics analysis

The two missense variants were predicted to be deleterious by both algorithms (Table 2).

### Evolutionary conservation analysis

The results obtained from HomoloGene showed that the amino acids corresponding to the two *DAB1* variants were highly conserved among different species (Table S3).

### Genetic Association Analysis

For our sample set of total cases (*n* = 2143) and controls (*n* = 2190), we computed a statistical power of >80% using the following parameters: disease prevalence of 0.01, high-risk allele frequency of 0.00088, genotypic relative risk Aa of ≥4.2, and type I error rate of 0.05. Genotypes were determined by TaqMan assays for 2079/2143 (97.0%) cases and 2154/2190 (98.4%) controls for p. G382C and 2076/2143 (96.9%) cases and 2137/2190 (97.6%) controls for p.V129I. The results of the genetic association analysis of missense mutations are shown in Table 3. We detected an additional G382C mutation carrier in the ASD group but none in the CON group. We also detected V129I in three SCZ samples and four CON samples. Although neither variant showed a significant difference in prevalence between cases and controls, G382C was not detected in our CON group or in any public databases (gnomAD, ESP, jMorp, HGVD).

**Table 3:** Association analysis of two rare missense mutations.

Variant	Genomic data		Cases (SCZ + ASD)				Control	
	Position^a^	M/m	Genotype count^b^	MAF	*P*-value^c^	Odds ratio	Genotype count^b^	MAF
G382C	1:57480856	C/A	0/1/2078	0.00024	0.49	3.11	0/0/2154	0
V129I	1:57538009	C/T	0/3/2073	0.00072	0.52	0.77	0/4/2133	0.00093

*SCZ* schizophrenia, *ASD* autism spectrum disorders, *M* major, *m* minor, *MAF* minor allele frequency.

^a^Genomic position based on NCBI build GRCh 37.p13 (Transcript ID ENST00000371236.2).

^b^Genotype count; homozygote of minor allele/heterozygote/homozygote of the major allele.

^c^P-values were calculated with Fisher’s exact test (2 × 2 contingency table, one-tailed).

### Phenotypic analysis

Detailed descriptions of the clinical information of the two cases with G382C are shown in Table S4. These cases did not seem to share any clinical symptoms.

### Protein three-dimensional (3D) structure analysis

**Fig. 2:**
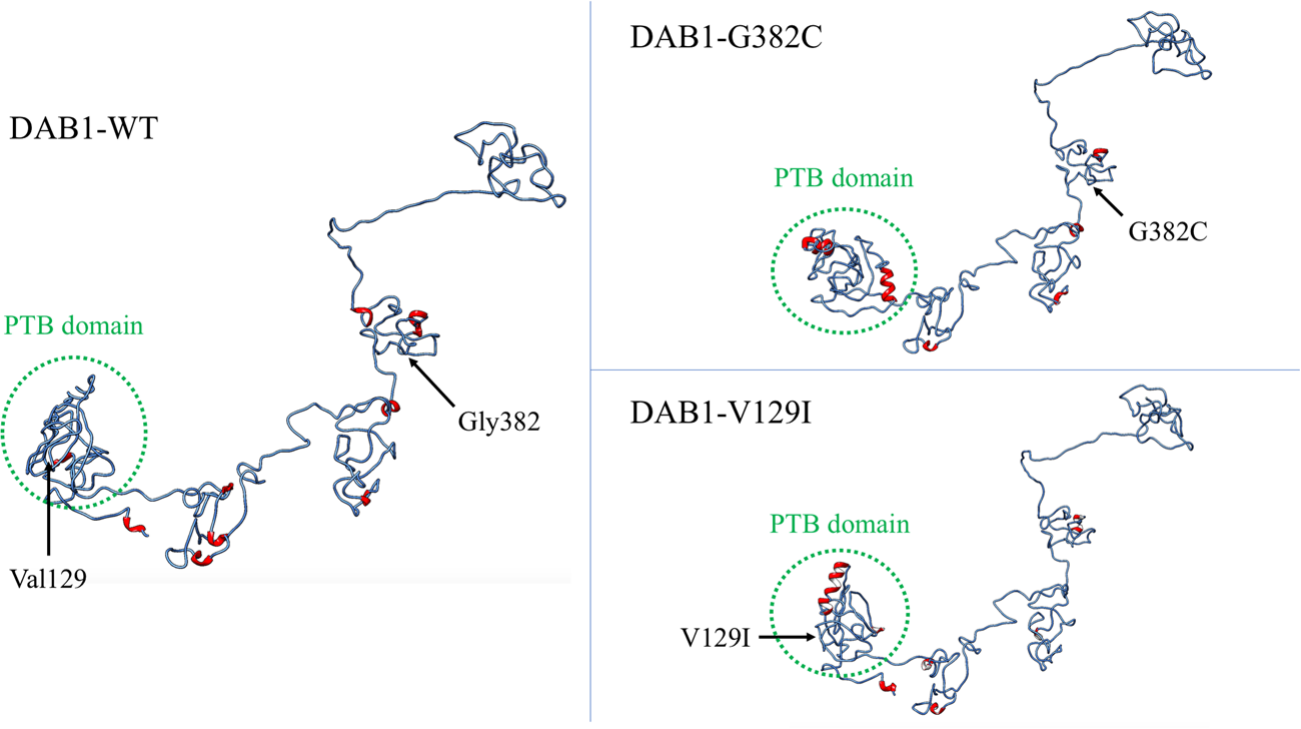
3D model of the protein structure of DAB1 with variants compared to the wild type. The protein structure of DAB1 is composed of a phosphotyrosine-binding (PTB) domain (green dotted circles) and a disordered C-terminus. Val129 and Gly382 are the 129th and 382nd residues of DAB1, respectively. The 3D modeling of mutated proteins with I-TASSER indicated that DAB1-G382C and DAB1-V129I could each change the protein structure. α-helices are marked in red.

The 3D modeling of the WT and mutated proteins with I-TASSER indicated that DAB1-G382C and DAB1-V129I could each change the protein structure (Fig. 2).

### CHX Chase Assay

**Fig. 3:**
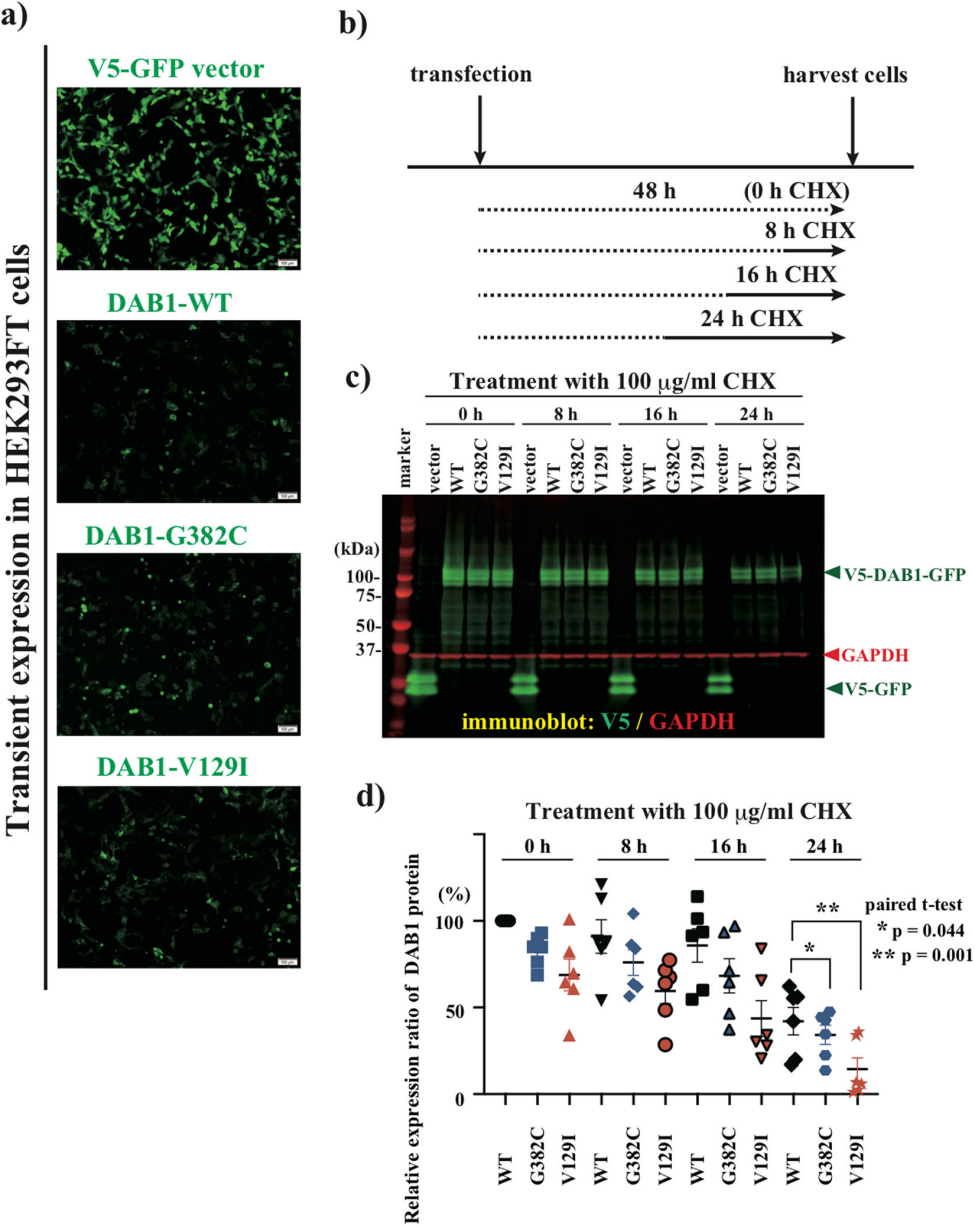
Cycloheximide chase assay of DAB1 transiently expressed in HEK293FT cells to assess biological stability. (**a**) Expression of *DAB1* plasmids in HEK293FT cells. Wild type (WT) and variant (G382C, V129I) DAB1 fused with both the V5-epitope tag and GFP at the C-terminus of the DAB1 protein. (**b**) Design of the cycloheximide (CHX) chase assay. The cells were harvested at 48 h after transfection and treatment with CHX during the last 8, 16, or 24 h. (**c**) Immunoblot quantitating the relative expression of V5 signals associated with DAB1. (**d**) Graphs showing the relative amount of DAB1 in six independent CHX chase assays. Six independent experiments were performed. The signal ratio of V5/GAPDH was plotted for each trial, and the mean and standard deviation were calculated.

For further functional analysis of DAB1 variants, we generated mammalian expression plasmids of DAB1-WT and variants (G382C, V129I) fused with both the V5-epitope tag and green fluorescent protein (GFP) at the C-terminus. We transfected these plasmids into HEK293FT cells and observed the fluorescence. We found little difference in intracellular localization among DAB1-WT and its variants (Fig. 3a). Then, we transfected these DAB1 expression plasmids into cultured cells and harvested the cells 48 h after transfection without or with CHX treatment during the last 8, 16, or 24 h (Fig. 3b). In six independent transfections and CHX treatment assays, both DAB1-G382C and DAB1-V129I variants showed a significant decrease in the DAB1 protein level compared to DAB1-WT in quantitative immunoblot analysis (Fig. 3c, d; Figure S1), suggesting that both mutant proteins are less stable than WT DAB1 protein.

## Discussion

To our knowledge, this is the first study to investigate the contribution of rare *DAB1* variants to neurodevelopmental disorders, including SCZ and ASD, and susceptibility to these disorders. We conducted mutation screening of exons in *DAB1* in 562 Japanese patients with SCZ or ASD and detected two SNVs. Two SNVs (G382C and V129I) were selected because they were present at a very low frequency in public databases and were predicted to be deleterious by *in silico* analyses. Association analysis was then performed with a cohort comprising 2143 cases and 2190 controls. Although several similar studies have identified a statistical association between rare SNVs and neurodevelopmental disorders^14,15^, we found no statistically significant association between the two rare heterozygous *DAB1* SNVs we investigated in this study and SCZ or ASD.

DAB1-G382C was detected only in our case samples and not in our CON group or any public databases (gnomAD, ESP, jMorp, HGVD). Recent large-scale genetic studies have reported that ultrarare and unique missense mutations are highly enriched in SCZ, especially in sets of genes with functions closely involved in brain function^51^. G382C was predicted to be deleterious by *in silico* analytic methods (PolyPhen-2 and SIFT). G382C is located in the C-terminal region of DAB1. Although the structure and function of this region have not been investigated in detail, the C-terminus may affect the strength of Reelin-DAB1 signaling by reducing DAB1 protein stability in specific neurons^52^. Analysis of the 3D structure of DAB1 suggested that G382C could change the protein structure. Furthermore, the CHX chase assay suggested that the DAB1-G382C mutant protein is more unstable than the DAB1-WT protein. Taken together, these results indicate that G382C may affect neuronal migration and synaptic plasticity through changes in Reelin-DAB1 signaling.

DAB1-V129I is located in the PTB domain of DAB1. PTB domains bind specifically to transmembrane proteins containing an NPXY internalization signal, such as lipoprotein receptors, and play an important role in Reelin-DAB1 signaling^53^. However, V129I was observed in three SCZ samples and four CON samples through association analysis. Detection of a significant association with DAB1-V129I in neurodevelopmental disorders such as SCZ and ASD will be difficult.

### Limitations

Several limitations should be considered when interpreting the results of our study. First, we screened for mutations in 370 SCZ and 192 ASD cases. Other rare SNVs may exist but not be detected in this study. Furthermore, because our sequencing was focused exclusively on the coding regions, we may have missed important mutations in regulatory regions and potential disease-associated regions such as the promoter, untranslated regions, or intronic regions of *DAB1*. We did not focus on CNVs because of the difficulty in detecting CNVs in this study. In our former CNV study using array comparative genomic hybridization with 2458 SCZ and 1108 ASD samples, including the samples used in this study, we did not identify any CNVs in the *DAB1* regions^44^. Second, we could not fully conduct segregation analyses for mutations due to limited access to the subjects’ family members. This made accurate measurement of the inheritance of mutations in the pedigree impossible. Furthermore, in the phenotypic analysis of cases with novel rare mutations, the effect of discovered rare variants could not be fully evaluated because we could not obtain detailed clinical information regarding the patients’ developmental period. Third, detailed structural analysis and functional assays of G382C were not conducted because the location of the mutation in the C-terminal region makes structure prediction difficult. We demonstrated that the two discovered SNVs reduced DAB1 protein stability. However, in a future study, we need to evaluate the potential impacts of the discovered variants on the pathophysiology of SCZ and ASD by investigating neurons in the brains of knock-in mice. Finally, *post hoc* calculations based on the minor allele frequency of association analysis showed that a much larger sample size will be required to reveal relationships between neurodevelopmental disorders and DAB1-G382C. In other words, because the common variant odds ratio detected by genome-wide association studies that focused on psychiatric disorders is approximately 1.2^5^ and because our study has sufficient power to rule out a lack of association only for variants with an odds ratio of 4.2 and higher, much larger studies will need to be conducted to comprehensively evaluate the potential association between SNVs in *DAB1* and neurodevelopmental disorders, especially for variants with odds ratios between 1.2 and 4.2.

## Conclusions

We sequenced the exons of *DAB1* in Japanese SCZ and ASD patients and discovered two rare missense variants that may increase susceptibility to SCZ and ASD. Although statistical significance was not detected, G382C was found only in case of SCZ or ASD. Moreover, G382C was suggested to change the protein structure of DAB1 and to reduce protein stability. Further research is needed using a much larger sample size for a more comprehensive evaluation of *DAB1*, along with genes in *DAB1*-related pathways.

## Supplementary information

Supplementary Information
