# Supplementary material for: Rare single-nucleotide DAB1 variants and their contribution to Schizophrenia and autism spectrum disorder susceptibility
Source: Hum Genome Var. 2020 Nov 10;7:37. doi: 10.1038/s41439-020-00125-7 (PMC7655853; doi:10.1038/s41439-020-00125-7)
Supplement: Supplementary file 1 — Supplementary Information [file 41439_2020_125_MOESM1_ESM.docx]

***Supplementary Information***

***Rare Single-Nucleotide DAB1 Variants and Their Contribution to Schizophrenia and Autism Spectrum Disorder Susceptibility***

Yoshihiro Nawa^1^, Hiroki Kimura^1*^, Daisuke Mori^1,2^, Hidekazu Kato^1^, Miho Toyama^1^, Sho Furuta^1^, Yanjie Yu^1^, Kanako Ishizuka^1^, Itaru Kushima^1,2^, Branko Aleksic^1^, Yuko Arioka^1,2,3^, Mako Morikawa^1^, Takashi Okada^1^, Toshiya Inada^1^, Kozo Kaibuchi^5^, Masashi Ikeda^6^, Nakao Iwata^6^, Michio Suzuki^7^, Yuko Okahisa^8^, Jun Egawa^9^, Toshiyuki Someya^9^, Fumichika Nishimura^10^, Tsukasa Sasaki^11^, and Norio Ozaki^1^

^1^ Department of Psychiatry, Nagoya University Graduate School of Medicine, Nagoya, Aichi, Japan

^2^ Brain and Mind Research Center, Nagoya University, Nagoya, Aichi, Japan

^3^ Institute for Advanced Research, Nagoya University, Nagoya, Aichi, Japan

^4^ Center for Advanced Medicine and Clinical Research, Nagoya University Hospital, Nagoya, Aichi, Japan

^5^ Department of Cell Pharmacology, Nagoya University Graduate School of Medicine, Nagoya, Aichi, Japan

^6^ Department of Psychiatry, Fujita Health University School of Medicine, Toyoake, Aichi, Japan

^7^ Department of Neuropsychiatry, University of Toyama Graduate School of Medicine and Pharmaceutical Sciences, Toyama, Japan

^8^ Department of Neuropsychiatry, Okayama University Graduate School of Medicine, Dentistry and Pharmaceutical Sciences, Okayama, Japan

^9^ Department of Psychiatry, Niigata University Graduate School of Medical and Dental Sciences, Niigata, Japan

^10^ Office for Mental Health Support, Center for Research on Counseling and Support Services, The University of Tokyo, Tokyo, Japan

^11^ Department of Physical and Health Education, Graduate School of Education, The University of Tokyo, Tokyo, Japan

*Corresponding author:

Hiroki Kimura, MD, PhD

Assistant Professor

Department of Psychiatry, Nagoya University Graduate School of Medicine

65 Tsurumai-cho, Showa-ku, Nagoya, Aichi-ken 466-8560, Japan

Tel: +81 52 7442282; Fax: +81 52 7442293

E-mail: kimura.hiroki@med.nagoya-u.ac.jp

**Contents of Supplementary Materials**

**Table S1. Primer sequences for validating each variant**

**Table S2. Details of detected variants in *Dab1* coding exons**

**Table S3. Results of conservation analysis**

**Table S4. Clinical information of carriers of the DAB1-G382C mutation**

**Figure S1. Expression of DAB1-V5-GFP plasmids in HEK293FT cells was confirmed with immunoblotting using an anti-DAB1 antibody.**

**Table S1. Primer sequences for validating each variant**

**Table S2. Details of detected variants in *Dab1* coding exons**

Abbreviations: Chr, Chromosome; SCZ, schizophrenia; ASD, autism spectrum disorders; MAF, minor allele frequency

^a^ Genomic position based on NCBI build GRCh 37 (Transcript ID ENST00000371236.2)

^b^ minor allele count/total allele count

^c^ Call quality is on a range 0 to 255

**Table S3. Results of conservation analysis**

Multiple sequence alignment of DAB1 protein obtained from HomoloGene. These alignments include the novel mutations.

**Table S4. Clinical information of carriers of the DAB1-G382C mutation**

Note: +; present, -; absent; NA; not available, IQ; Intelligence Quotient

**Figure S1. Expression of DAB1-V5-GFP plasmids in HEK293FT cells was confirmed with immunoblotting using an anti-DAB1 antibody.**
